# Hyaluronic Acid Dermal Filler for Inducing Mechanical Ptosis in Facial Nerve Palsy: A Novel Approach to Treat Exposure Keratopathy

**DOI:** 10.22336/rjo.2025.06

**Published:** 2025

**Authors:** Neeraj Sharma, Vipin Rana, Amit Nandan Tripathi, Kalpamoi Kakati

**Affiliations:** Department of Ophthalmology, Command Hospital, (Eastern Command), Kolkata, India

**Keywords:** dermal filler, hyaluronic acid, lagophthalmos, facial nerve palsy, HA = Hyaluronic acid, OSDI = Ocular Surface Disease Index

## Abstract

**Background:**

This study aimed to assess a dermal filler’s safety, efficacy, and outcome for inducing temporary mechanical ptosis of the upper eyelid in patients with facial nerve palsy with exposure keratopathy.

**Methods:**

A prospective interventional study was conducted on 13 patients with facial nerve palsy with various levels of lagophthalmos and exposure keratopathy. A total of 0.3 ml of dermal filler (Juvéderm Ultra Plus XC, Allergan, USA) was injected subdermally over the pretarsus area of the upper lid to induce mechanical ptosis. Post-dermal filler implantation patients were followed up for a reduction in the amount of lagophthalmos and adverse effects.

**Results:**

Out of 13 patients exposed to keratopathy due to facial nerve palsy, two were females, and eleven were males, with a mean age of 54+/-6.5 years and House Brackmann severity grades IV and V. The preinjection average lagophthalmos was 5.5 mm, and it decreased to an average of 0.8 mm postinjection at 1 week of follow-up, which was maintained at 12 weeks and 24 weeks of follow-up. No adverse side effects were observed during the 24-week follow-up.

Discussion: This study highlights the effectiveness and safety of hyaluronic acid-based dermal fillers in treating lagophthalmos and exposure keratopathy caused by facial nerve palsy. A single 0.3 ml injection in the pretarsal region successfully induced mechanical ptosis, significantly reducing lagophthalmos and protecting the cornea, with results sustained for 24 weeks and no adverse effects. Offering a minimally invasive, cost-effective alternative to traditional surgical methods, dermal fillers show promise, particularly in early-stage management. Despite a small sample size and limited follow-up, these findings pave the way for future research to refine dosing and assess long-term efficacy.

**Conclusions:**

Absorbable dermal filler implants are an easy and effective method for inducing mechanical ptosis and protecting the cornea in patients with exposure keratopathy due to facial nerve palsy.

## Introduction

Facial nerve palsy (both upper motor and lower motor neuron types) can affect the closure of the lid, leading to epiphora, grittiness in the eye, and a risk of corneal exposure. Common causes of facial nerve palsy are stroke, hemorrhage in the pons, infection of the middle ear (Ramsay-Hunt syndrome), surgeries involving the middle ear, Bell’s palsy, nasopharyngeal carcinoma, facial trauma, and parotid gland surgeries [1]. This risk is aggravated in individuals with poor Bell’s phenomenon [2]. Corneal exposure can lead to exposure keratopathy, and superadded corneal infection can lead to dreaded complications such as infective keratitis and vision loss [3].

Traditionally, all patients with lagophthalmos and exposure keratopathy are managed with the liberal use of artificial tear substitutes, night-time lid taping, temporary or permanent tarsorrhaphy, botulinum toxin injection in the upper lid, and gold weight implantation in the upper lid to induce mechanical ptosis [4].

Dermal fillers are artificially manufactured complex polysaccharides that dermatologists and cosmetic surgeons have used to rejuvenate the face as a day-care procedure. Injectable fillers can reshape the jawline, lift soft tissues, and improve facial proportions [5,6]. Hyaluronic acid (HA) is the most common dermal filler used. Due to its water affinity, it improves volume loss by filling, volumizing, and hydrating the injected area. Otorhinolaryngologists have also used these agents to lateralize vocal cords in vocal cord palsy [7,8]. Given the widespread use of dermal fillers, this study was conducted to induce mechanical ptosis by injecting these fillers into the pretarsal area of the upper lid in patients with exposure keratopathy due to facial nerve palsy.

## Materials and methods

### Study Design

This prospective interventional study was conducted at a tertiary care hospital in eastern India from January 2023 to December 2023. The hospital’s local ethical committee approved the study per the principles of the Declaration of Helsinki. The research was registered with the Clinical Trial Registry of India (CTRI) with the registration number CTRI/2023/01/049176.

The inclusion criteria were patients diagnosed with facial nerve palsy, irrespective of its etiology, and who demonstrated lagophthalmos. Both permanent and temporary causes of facial nerve palsy, such as Bell’s palsy and stroke-related causes, were included to examine the adaptability of the dermal filler approach for managing lagophthalmos. The severity of exposure keratopathy due to facial nerve palsy was based on the House-Brackmann grading system [9]. Patients with lagophthalmos resulting from causes other than facial nerve palsy, like cicatricial ectropion, chemical or thermal injuries, orbital disorders, and traumatic lid avulsion, were excluded.

### Primary Objective

**To estimate the degree of change in lagophthalmos (measured in millimeters)** after dermal filler delivery in patients with facial nerve palsy. Measurements were to be taken on day one, at the end of week one, and at the end of the 4-week, 12-week, and 24-week follow-ups to measure the immediate and long-term effects of the treatment.

### Secondary Objectives

**To determine** improvement in forceful lid closure, the ability of patients to achieve and maintain forceful eyelid closure after therapy was assessed, focusing on the amount and duration of any residual upper eyelid droop.

**To determine changes in corneal status**, the cornea was examined before and after the procedure and during one-week, 12-week, and 24-week follow-up appointments for changes such as Dellen formation and punctate epithelial erosions.

**The ocular surface disease index (OSDI) was used to quantitatively track the progression of patients’ ocular symptoms over 24 weeks to estimate changes in ocular symptoms**.

****To determine dermal filler-related changes, we documented any palpable changes at the injection site during the 24-week follow-up period****, emphasizing nodular swelling or other substantial alterations.

**To determine the incidence of adverse reactions**, monitor and document any severe reactions to dermal fillers to assess the treatment’s safety profile.

### Procedure

A cohort of 13 patients who presented with facial nerve palsy and lagophthalmos was identified. Detailed medical histories were extracted to ascertain the etiology and duration of the palsy. A thorough ophthalmic examination comprised best-corrected visual acuity using Snellen’s charts, exposure keratopathy signs determined by corneal staining with fluorescein dye, Bell’s phenomenon evaluation, and an appraisal of corneal sensations. The extent of lagophthalmos was determined using a slit lamp and confirmed in the operation theatre with patients placed in a supine position. Measurements were recorded using a surgical scale. A 0.3 ml preloaded dermal filler injection (Juvéderm Ultra Plus XC, Allergan, USA) containing 24 mg/ml hyaluronic acid combined with 0.3% w/w physiological buffer was administered. Under strict aseptic measures in the operating room, the upper lid underwent subdermal filler injection in the pretarsal region using a 26-gauge needle located two mm from the lid margin postinjection, and patients were supervised in the operation theatre for possible allergic reactions to the filler.

### Statistical Methods

The data were recorded with an MS Excel spreadsheet. SPSS v23 (IBM Corp.) was used for data analysis. Descriptive statistics were presented as the means/standard deviations and medians/IQRs for continuous variables and as frequencies and percentages for categorical variables.

## Results

### Patient Demographics and Baseline Information (Table 1, 2)

There were 13 patients, 2 of whom were females and 11 of whom were males, with a mean age of 54+/-6.5 years. Bell’s palsy was diagnosed in four patients (30%). Two patients (15% each) had facial nerve palsy due to brain stroke and post-neurosurgical intervention for brain tumors. Additionally, two patients (15%) experienced palsy post-head and neck surgeries. The remaining three cases (23%) of facial nerve palsy resulted from head injury, antitubercular therapy for lung tuberculosis, and metastatic carcinoma of the lung. According to the House Brackmann scale, seven patients (53%) were Grade IV, and the remaining six (47%) were Grade V. All participants reported eye-related discomfort, watering, and a gritty eye sensation. The majority of patients maintained a visual acuity of 20/20 at presentation. However, one patient had compromised visual acuity due to corneal opacity and optic atrophy from a brain tumor. Two patients with Bell’s palsy displayed dellen formation on the temporal aspect of the cornea. Fluorescein dye staining revealed punctate staining in all participants’ lower third of the cornea.

**Table 1:** Patient demographics and clinical characteristics

S.No	Age	Sex	Cause of Facial Nerve Palsy	Onset	Vision at Presentation
1	45	F	Bell’s palsy	Acute	20/20 OU
2	52	M	Bell’s palsy	Chronic	20/20 OU
3	72	M	Post squamous cell carcinoma (operated)	Acute	20/20 OU
4	42	M	CP angle tumor (operated)	Chronic	Perception of light
5	58	F	Bell’s palsy	Acute	20/20 OU
6	64	M	Post stroke	Chronic	20/20 OU
7	34	M	CP angle tumor (operated)	Acute	20/20 OU
8	75	M	Post brain stroke	Chronic	20/20 OU
9	35	M	Bell’s palsy	Acute	20/20 OU
10	58	M	Carcinoma lung metastasis	Acute	20/20 OU
11	56	M	Post anti-tuberculosis therapy	Acute	20/20 OU
12	60	M	Post oropharyngeal carcinoma surgery	Acute	20/20 OU
13	23	M	Post head injury	Chronic	20/20 OU

**Table 2:** Clinical findings and lagophthalmos outcomes

Sr. No	Bell’s Phenomenon	Symptomatic/Corneal Staining	Lagophthalmos (1 Week)	Lagophthalmos (1 Month)	House-Brackmann) Grade
1	Good	Yes	Nil	Nil	IV
2	Fair	Yes/Dellen	Nil	Nil	IV
3	Fair	Yes	1	1	IV
4	Good	Yes/Corneal epithelial defect	1	1	V
5	Fair	Yes	Nil	Nil	IV
6	Fair	Yes	1	1	V
7	Fair	Yes	Nil	Nil	IV
8	Poor	Yes	2	2	V
9	Fair	Yes	Nil	Nil	IV
10	Fair	Yes	3	2	V
11	Fair	Yes	1	Nil	IV
12	Fair	Yes	2	2	V
13	Fair	Yes/Dellen	Nil	Nil	IV

### Primary outcome

**Fig. 1:**
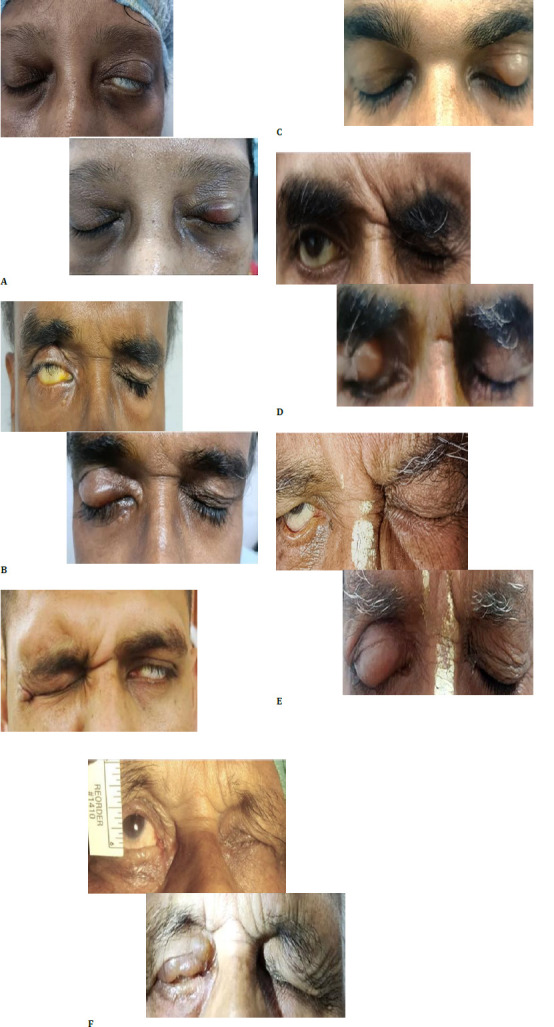
A-F Pre- and post-dermal filler images showing the reduction in lagophthalmos

Following dermal filler application, mechanical ptosis was evident in all patients, reaching its peak on day seven (**Fig. 1 A-F**). By this time, six patients (50%) had complete eyelid closure, which negated lagophthalmos. The remaining patients showed varying degrees of residual lagophthalmos: three had a length of 1 mm, two had a length of 2 mm, and one had a length of 3 mm. The median initial lagophthalmos was 5.5 mm (IQR: 4.5-6 mm), which decreased to 0.8 mm one week posttreatment (IQR: 0.5-1.2 mm).

### Secondary Outcomes

**Fig. 2:**
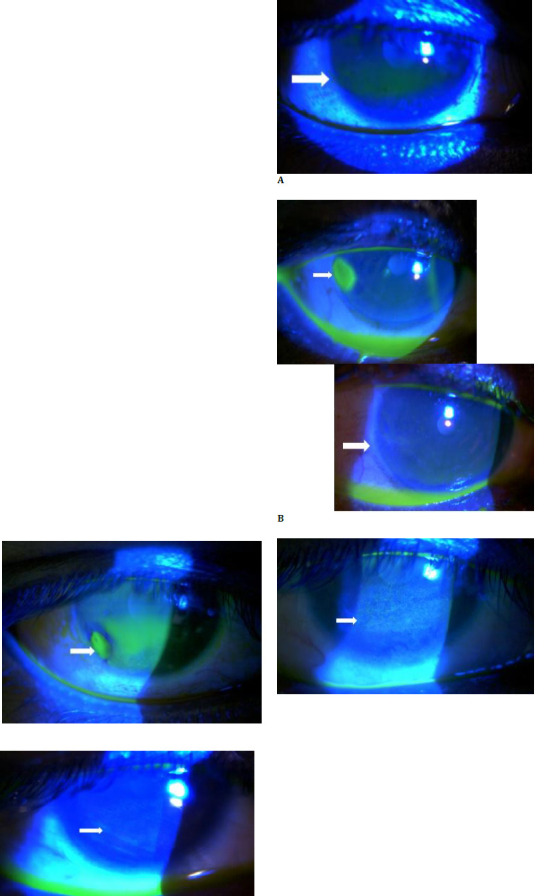
**A-C** Images showing improvement in exposure keratopathy post dermal filler injection (**A, B**) showing corneal dellen (white arrow) resolution and **C** showing improvement in inferior punctate staining (white arrow)

1. *Forced lid closure*: A brief drop of the upper eyelid commenced upon forceful closure, causing lid closure within 2-3 minutes and significantly reducing the palpebral fissure height and lagophthalmos.
2. *Corneal Status*: On initial examination, two patients with Bell’s palsy had evident dellen formation on the cornea’s temporal side (**Fig. 2 A, B**). Fluorescein dye staining revealed punctate staining in all patients (**Fig. 2 C**). By 1 week, all dellen formations and punctate epithelial erosions had resolved, indicating the rapid therapeutic potential of the filler treatment. This improvement persisted throughout the 24-week observation.
3. *Ocular Surface Disease Index (OSDI) Assessment*: Pretreatment OSDI scores, which averaged 45, indicated considerable discomfort. Following treatment and alignment with the resolution of corneal issues, a significant decrease in OSDI scores was observed. By the end of week one, the average score reached approximately 5, denoting minimal discomfort, and remained consistent throughout the 24-week timeframe.
4. *Dermal Filler Observations*: After the procedure, palpable nodular swelling was evident in the upper eyelid, which persisted for the entire 24-week monitoring duration. Fortunately, severe adverse reactions such as anaphylaxis or vascular embolisms that could impair visual clarity were not observed.

## Discussion

In our study, lagophthalmos in all thirteen patients was adequately reduced after receiving 0.3 ml of dermal filler containing hyaluronic acid (Juvéderm Ultra Plus XC, Allergan, USA) in the pretarsal region of the upper eyelid. The clinical response persisted through the final 24-week follow-up without any adverse reactions. Mechanical ptosis was most prominent at the end of the first week. The average lagophthalmos at one week decreased from 5.5 mm preinjection to 0.8 mm on average one week after the injection, and this response continued throughout the 24-week follow-up period. The decrease in lagophthalmos and nighttime corneal protection thus helped protect the cornea and reduce the signs and symptoms of exposure keratopathy.

Dermal fillers have been used to rejuvenate the face’s appearance for years. They are increasingly employed as a nonaesthetic alternative to standard surgical techniques for eyelid malposition and orbital volume insufficiency [10]. Hyaluronic acid injections have been utilized to alleviate lagophthalmos caused by superior sulcus deformity and to improve enophthalmos [11].

This study examined the efficacy of hyaluronic acid gel injections for treating paralytic lagophthalmos. Only two studies were found via a literature search in PubMed, Medline, Embase, and Scopus using the terms “exposure keratopathy” and “hyaluronic acid”, yielding limited evidence. In their retrospective study of nine patients (10 eyelids) treated with 0.9 ml of hyaluronic acid gel in the prelevator aponeurosis or pretarsal region, Mancini et al. reported a mean improvement of 4.5 mm in lagophthalmos. In contrast, our prospective study administered a single injection of a lower dose (0.3 ml) of dermal filler in the pretarsal region [12]. Despite the reduced dosage, all our patients experienced a significant reduction in lagophthalmos, eliminating corneal exposure during the 24-week follow-up period. Furthermore, another retrospective study by Martín-Oviedo et al., comprising 26 patients, used variable doses of dermal filler ranging from 0.3 ml to 1 ml and achieved a good response [13]. Martín-Oviedo’s study noted that 20% of patients did not respond adequately and required additional dermal filler injections or platinum implants. However, in our research, we did not require any additional dermal filler, and adequate corneal protection was achieved in all patients over a follow-up period of 24 weeks; however, the follow-up period in our study was shorter than the 1 year of follow-up reported by Martín-Oviedo et al. Thus, our findings suggest that a fixed dose of 0.3 ml of hyaluronic acid gel in the pretarsal region can be a highly effective treatment for reducing lagophthalmos, resulting in favorable outcomes and minimal complications.

Our study underlined that hyaluronic acid injections were most beneficial when administered during the earliest stages of lagophthalmos, such as in patients with Bell’s palsy and acute head injury. We hypothesized that the following factors may be involved. First, due to chronic lagophthalmos and extended inactivity, the afflicted eyelid muscles may atrophy. Second, even in acute lagophthalmos, the surrounding tissue, such as the skin and underlying tissues, may be somewhat flexible and supportive. Third, persistent lagophthalmos may cause changes in the tissue around the eyes and continuing inflammation, reducing the efficacy of the filler.

**Fig. 3:**
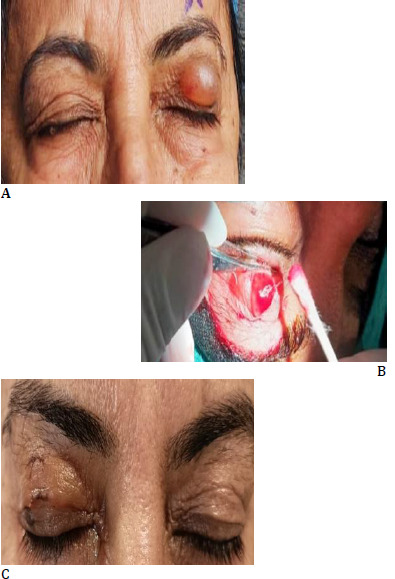
**A-C** Image **A** shows an enlarged nodule following dermal filler, **B** shows the expression of the dermal filler with a cotton bud following a 2 mm incision over the most prominent part of the nodule, and **C** shows the standard color and texture of the skin after dermal filler removal

Dermal fillers made of hyaluronic acid have many advantages. As dermal filler can be stored in a refrigerator, a single prefilled syringe containing one ml of dermal filler was used to inject 0.3 ml of dermal filler into the upper eyelid of each of the three patients; thus, this approach was less expensive than the use of gold or platinum implants, which are standard procedures used for exposing keratopathy, especially in chronic patients. However, the longevity of this procedure for inducing mechanical ptosis compared with gold and platinum implants needs to be studied. Their easy administration and removal also enhance the utility of dermal filler implants once the patient has recovered from facial nerve palsy. It is appropriate for people who are poor surgical candidates or who have mental instability. Because it is a less invasive procedure than tarsorrhaphy and gold/platinum implants, problems such as hematomas, infections, and lid margin distortion are not noted. The nodular swelling in the upper eyelid may raise some cosmetic concerns. However, it is a reversible procedure wherein dermal filler can be removed with a small skin incision of 2 mm and expressed using a cotton bud (**Fig. 3 A-C**) or by administering an injection of hyaluronidase.

The limitations of our study, including its small sample size, lack of a control group, and short follow-up period of 24 weeks, should be considered when interpreting the findings. Despite its limitations, this study provides valuable insights into the potential benefit of dermal filler implantation in managing lagophthalmos associated with facial nerve palsy. The inclusion of various causes of facial nerve palsy and rigorous adherence to ethical standards enhance the study’s credibility. The objective measurement of lagophthalmos and the absence of significant adverse reactions to the dermal filler add to the study’s strengths.

## Conclusion

This study highlighted and reaffirmed the role of dermal fillers in patients with exposure keratopathy caused by facial nerve palsy. Their use is simple, efficient, safe, and affordable. Nonophthalmic clinicians, particularly in intensive care settings, can easily use this agent to treat exposure keratopathy in comatose patients. Thus, this study added to the existing armamentarium for treating exposure keratopathy. However, further studies are needed to titrate the dose of dermal filler based on the amount of lagophthalmos and the type of facial nerve palsy (temporary/permanent). A long-term follow-up with these patients will reveal the longevity of this effect and the necessity for additional augmentation with the same or another type of dermal filler.
